# N-Carbamylglutamate Enhances Pregnancy Outcome in Rats through Activation of the PI3K/PKB/mTOR Signaling Pathway

**DOI:** 10.1371/journal.pone.0041192

**Published:** 2012-07-27

**Authors:** Xiangfang Zeng, Zhimin Huang, Xiangbing Mao, Junjun Wang, Guoyao Wu, Shiyan Qiao

**Affiliations:** 1 State Key Laboratory of Animal Nutrition, China Agricultural University, Beijing, China; 2 Departments of Animal Science and of Veterinary Integrative Biosciences, Texas A&M University, College Station, Texas, United States of America; The Ohio State Unversity, United States of America

## Abstract

Administration of N-carbamylglutamate (NCG), an analogue of endogenous N-acetyl-glutamate (an activator of arginine synthesis) has been shown to enhance neonatal growth by increasing circulating arginine levels. However, the effect of NCG on pregnancy remains unknown. This study examined the effects of NCG on pregnancy outcome and evaluated potential mechanisms involved. Reproductive performance, embryo implantation, and concentration of amino acids in serum and uterine flushing, were determined in rats fed control or NCG supplemented diets. Ishikawa cells and JAR cells were used to examine the mechanism by which NCG affects embryo implantation. Dietary NCG supplementation increased serum levels of arginine, onithine, and proline, as well as uterine levels of arginine, glutamine, glutamate, and proline. Additionally, it stimulated LIF expression, and enhanced the activation of signal transduction and activator of transcription 3 (Stat3), protein kinase B (PKB), and 70-kDa ribosomal protein S6 kinase (S6K1) during the periimplantation period, resulting in an increase in litter size but not birth weight. In uterine Ishikawa cells, LIF expression was also enhanced by treatment with arginine and its metabolites. In trophoblast JAR cells, treatment with arginine and its metabolites enhanced Stat3, PKB, and S6K1 activation and facilitated cellular adhesion activity. These effects were abolished by pretreatment with inhibitors of phosphatidylinositol 3-kinase (wortmannin) and mammalian target of rapamycin (rapamycin). The results demonstrate that NCG supplementation enhances pregnancy outcome and have important implications for the pregnancy outcome of mammalian species.

## Introduction

With unprecedented changes in economic and social development as well as advanced maternal age, low fertility rate has become a global concern [1], [2]. Early pregnancy loss is a common complication in human gestation [3] and reduced fertility in farm animals is of critical importance for efficient animal production [4]. The embryo implantation process is a key step in reproduction [5]. Embryonic loss of up to 50% occurs primarily during the pre-implantation or peri-implantation period for most mammals [6], [7]. Among the many factors that are involved in this process [8], leukemia inhibitory factor antibody (LIF) plays an essential role [9]. One of the initial events during embryo implantation is the adhesion of trophoblast cells to glycoproteins in the extracellular matrix of the uterine epithelium, such as fibronectin, vitronectin, and laminin [10]. LIF has been shown to promote extravillous trophoblast adhesion to fibronectin, vitronectin, and laminin during the first trimester in human pregnancy [11]. Stat3 is essential for embryo implantation, which can be activated by LIF [12].

Arginine has a great impact on embryonic/fetal survival, growth and development [13]. Dietary arginine supplementation can prevent fetal growth restriction in humans and rats [14], [15], increase the number of live-born piglets in sows/gilts [16], [17], and improve embryo implantation in rats [18]. N-carbamylglutamate (NCG), a structural analogue of N-acetylglutamate, which activates a key enzyme (carbamylphosphate synthetase-1) of the arginine-synthetic pathway [19], is used clinically in the treatment of N-acetylglutamate synthase deficiency, organic acidurias, and maple syrup urine disease [20]–[22]. NCG administration has been shown to stimulate citrulline and arginine synthesis in enterocytes [19] and increase endogenous synthesis of arginine, plasma concentrations of arginine and somatotropin, growth rate, and muscle protein synthesis in sow-reared piglets [19], [23]. Furthermore, dietary NCG supplementation can increase intestinal growth and heat shock protein-70 expression in weanling pigs [24]. At present, little is known about the effects of NCG on pregnancy. We hypothesized that dietary NCG supplementation during pregnancy can stimulate endogenous synthesis of arginine and increase the levels of arginine and its metabolites in serum and uterine fluids, thereby improving reproductive performance. The objective of the present study was to test this hypothesis in gestating rats, in Ishikawa and JAR cells to identify the mechanism involved.

## Results

### Reproductive performance of rats fed NCG-supplemented diet

Dietary NCG supplementation increased litter size, live-born pup number, litter birth weight, and litter birth weight of live-born rats, compared with the control group (*P*<0.001) (Table 1). There were no differences in birth weight or the ratio of female∶male rat pups among groups (Table 1). In comparison with 0.05% NCG supplementation, 0.1% NCG supplementation further increased litter size, live-born pup number, litter birth weight, and litter birth weight of live-born rats (*P*<0.001, Table 1).

**Table 1:** Reproductive performance of primiparous rats fed NCG supplemented or control diets during pregnancy*.

Items	Control	0.05% NCG	0.1% NCG	SEM	*P* value
N	96	96	96		
Litter size	11.6±0.23^a^	12.4±0.23^b^	13.1±0.15^c^	0.21	<0.001
Live-born rats, n/litter	11.4±0.23^a^	12.2±0.23^b^	13.0±0.15^c^	0.21	<0.001
Litter weight, g	73.2±1.44^a^	78.2±1.20^b^	83.3±1.09^c^	1.25	<0.001
Litter birth weight of live-born rats, g	72.6±1.42^a^	77.5±1.18^b^	82.8±1.16^c^	1.24	<0.001
Birth weight, g	6.34±0.03^a^	6.35±0.03^a^	6.34±0.02^a^	0.03	0.92
Birth weight of live-born rats, g	6.38±0.03^a^	6.37±0.03^a^	6.36±0.02^a^	0.03	0.85
Female/male	1.09±0.05^a^	1.07±0.06^a^	1.07±0.07^a^	0.07	0.97

* Data are means ± SEM. Means in a row without a common letter differ, *P*<0.05.

### Amino acid concentrations in maternal serum and uterine flushings of rats fed a NCG-supplemented diet

Serum concentrations of proline, ornithine, and arginine were increased in NCG-supplemented rats, compared with controls (*P*<0.05, Table 2). No changes in circulating levels of other amino acids were observed among the treatment groups (Information S1).

**Table 2:** Serum concentrations of amino acids in pregnant rats fed NCG-supplemented or control diets^1^.

	Treatment		
Amino acids	Control (µmol/L)	0.1% NCG (µmol/L)	*P* value
N	15	15	
Proline	181.2±12.8	218.1±8.7*	0.03
Ornithine	101.5±15.8	172.2±21.0*	0.01
Arginine	130.8±18.2	195.1±16.8*	0.01
Glutamate	206.0±16.5	250.9±27.5	0.17
Glutamine	417.1±26.4	447.4±33.7	0.48
Ammonia	335.9±29.7	279.7±14.2	0.11
Urea	634.3±25.2	584.5±36.0	0.27

1 Data are means ± SEM.

* *P*<0.05: Different from the control group.

The concentrations of proline, arginine, glutamate, and glutamine in uterine flushings of rats fed the NCG-supplemented diet were higher than in rats fed the control diet (*P*<0.05, Table 3). In contrast, urea concentration tended to decrease (*P*<0.07) in the NCG compared with the control group. Concentrations of other amino acids in uterine flushings did not differ among the treatment groups (Information S1).

**Table 3:** Amino acid concentrations in uterine flushings of pregnant rats fed NCG-supplemented or control diets^1^.

	Treatment		
Amino acids	Control (µmol/L)	0.1% NCG (µmol/L)	*P* value
N	15	15	
Proline	21.6±2.91	51.7±8.11*	0.003
Ornithine	1.48±0.25	1.57±0.27	0.86
Arginine	3.09±0.19	3.67±0.14*	0.03
Glutamate	36.6±3.9	49.1±8.30*	0.03
Glutamine	22.6±2.13	33.8±4.33*	0.03
Ammonia	29.9±2.16	32.9±3.14	0.25
Urea	26.0±2.66	18.7±1.48	0.07

1 Data are means ± SEM.

* *P*<0.05: Different from the control group.

### Intrauterine injection of LIF antibody, wortmannin, or rapamycin during pregnancy in rats fed a NCG-supplemented diet

**Figure 1:**
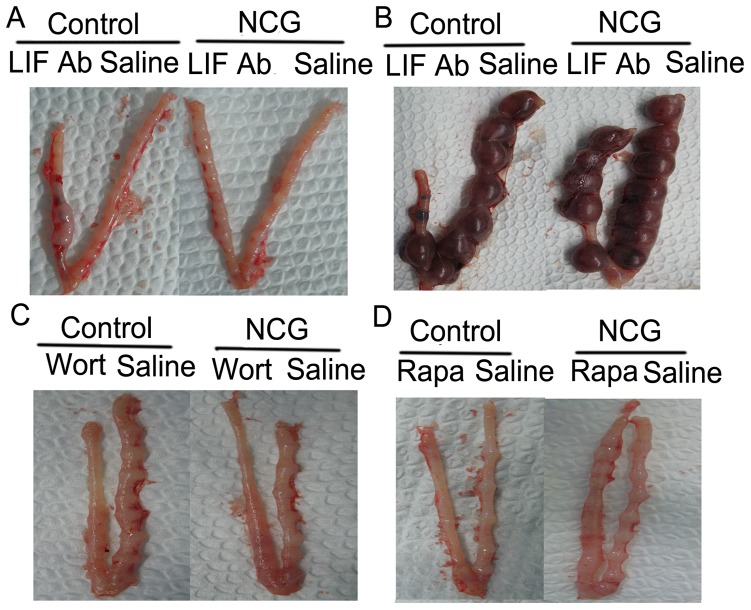
Embryo implantation in the presence of LIF antibody, wortmannin or rapamycin. Embryo implantation in the presence of LIF antibody on d 7 (A) and d 15 (B) of pregnancy, wortmannin on d 7 of pregnancy (C), or rapamycin on d 7 of pregnancy (D) in rats fed a 0.1% NCG-supplemented or control diets. Ab = antibody, Wort = wortmannin, Rapa = rapamycin.

Because LIF may be a novel factor mediating the beneficial action of NCG on pregnancy outcome, we determined the effects of LIF antibody on the location of embryo implantation sites on d 7 of pregnancy, as well as the number of viable fetuses, weights of conceptuses, fetuses, and placentas on d 15 of pregnancy in rats fed the control or NCG-supplemented diet. In rats fed the control diet, the implantation sites were well distributed in the control uterine horn on d 7 of pregnancy, but were crowded in rats receiving administration of the LIF antibody into the uterine horn (Fig. 1A). However, in rats fed the NCG supplemented diet, the implantation sites were well spaced both in the control and the LIF antibody-treated uterine horn (Fig. 1A). On d 15 of pregnancy, obvious embryo resorption was observed in the uterine horn of rats receiving the LIF antibody but not in rats fed the NCG-supplemented diet (Fig. 1B).

**Figure 2:**
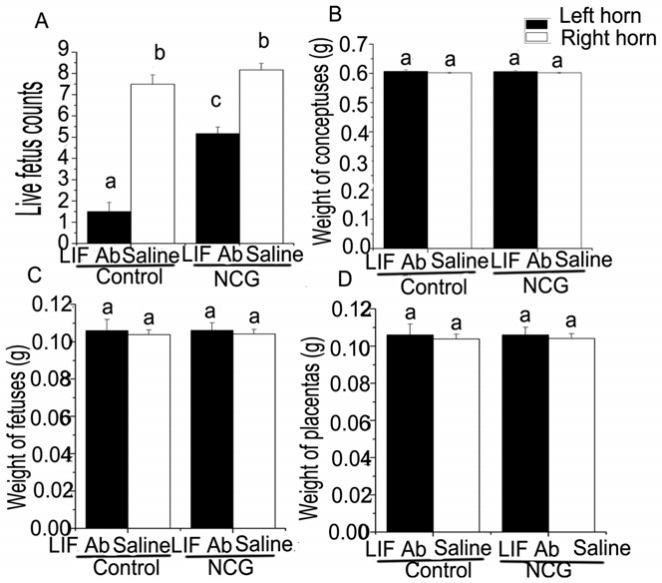
Live fetus counts and the weight of conceptuses, fetuses, and placentas of rats with or without intrauterine injection of LIF antibody. Live fetus counts (A), weight of conceptuses (B), fetuses (C), and placentas (D) on d 15 of pregnancy in the presence and absence of LIF antibody in rats fed 0.1% NCG-supplemented or control diets. Data were given as means ± SEM, n = 6. Means with different letters were significant (*P*<0.05). Ab = antibody.

The number of live fetuses on d 15 of pregnancy was comparable in the right uterine horns without administration of the LIF antibody between the control group and NCG group (Fig. 2A). However, the number of live fetuses was lower in the LIF antibody-treated uterine horns in rats fed the control diet, compared with rats fed the NCG-supplemented diet (*P*<0.05, Fig. 2A). No differences in weights of conceptuses (Fig. 2B), fetuses (Fig. 2C), and placentas (Fig. 2D) on d 15 of pregnancy were detected between the control and NCG groups, or between the LIF antibody-treated and non-treated uterine horns.

Intrauterine administration of wortmannin, the inhibitor of phosphatidylinositol 3-kinase (PI3K), completely inhibited embryo implantation in rats fed the control or the NCG-supplemented diet (Fig. 1C). A similar response was observed in rats fed the control diet which received intrauterine administration of rapamycin, the inhibitor of mammalian target of rapamycin (mTOR) (Fig. 1D). However, in rats fed the NCG-supplemented diet, embryo implantation was not totally inhibited, although the number of implanted embryos was reduced (Fig. 1D).

### Uterine expression of LIF and phosphorylated protein kinase B (p-PKB), phosphorylated 70-kDa ribosomal protein S6 kinase (p-S6K1), and phosphorylated signal transduction and activator of transcription 3 (p-Stat3) in rats fed a NCG-supplemented diet

**Figure 3:**
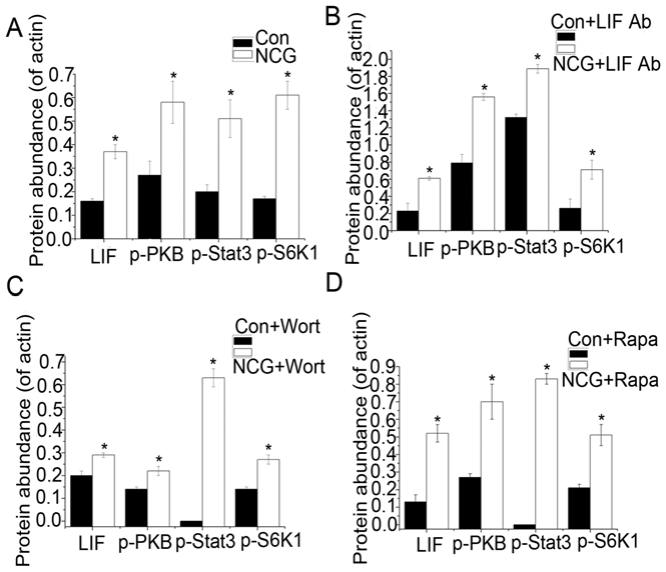
The relative protein abundance of uterine p-Stat3, LIF, p-S6K1, and p-PKB. The relative protein abundance of uterine p-Stat3, LIF, p-S6K1, and p-PKB in rats fed a NCG-supplemented or control diet on d 5 (A), and d 7 of pregnancy in the presence of LIF antibody (B), and on d 7 in the presence of wortmannin (C) and rapamycin (D). Con = Control, Ab = antibody, Wort = wortmannin, Rapa = rapamycin.*means significance in comparison with the control.

Dietary NCG supplementation increased (*P*<0.05) uterine expression of LIF, p-Stat3, p-PKB, and p-S6K1 by 1.32-fold, 1.56-fold, 1.12-fold, and 3.30-fold, respectively, compared with the control group on day 5 of pregnancy (Fig. 3, Information S2). In response to intrauterine administration of the LIF antibody on day 4 of pregnancy, the expression of LIF and p-PKB, p-S6K1, and p-Stat3 was also increased (*P*<0.05) by 1.64-fold, 0.96-fold, 1.67-fold, and 0.43-fold in NCG group on d 7 of pregnancy, compared with controls (Fig. 3, Information S2).

In the control group, intrauterine administration of wortmannin markedly attenuated the expression of uterine LIF and p-S6K1. The expression of Stat3 appeared to be undetectable whereas p-PKB was detectable but significantly decreased. In the NCG group, the expression of LIF, p-PKB, p-S6K1, and p-Stat3 was enhanced in the uterus (Fig. 3, Information S2). In response to intrauterine administration of rapamycin, the expression of LIF, p-S6K1, and p-Stat3 in the uterus of the control group was weak. However, in the NCG group, the expression of uterine LIF, p-PKB, p-S6K1, and p-Stat3 was increased (Fig. 3, Information S2).

### Expression of p-PKB, p-S6K1, and p-Stat3 in JAR cells in response to arginine, glutamine, glutamate, and proline

Because the concentrations of arginine, glutamine, glutamate, and proline in uterine flushing were increased in response to dietary NCG supplementation, we conducted a series of experiments using JAR cells and Ishikawa cells as *in vitro* models to determine the effects of these four amino acids on embryo implantation. The expression of p-PKB, p-S6K1, and p-Stat3 was increased (*P*<0.05) in JAR cells treated with arginine (Information S2) and glutamine (Information S2) in a dose-dependent manner (Table 4). Proline (Information S2) also activated the expression of p-PKB and p-S6K1, but glutamate did not (Table 4, Information S2). p-Stat3 was not detectable in cells treated with proline and glutamate. Arginine increased the expression of p-PKB, p-S6K1, and p-Stat3 within 0.5 h (Table 5, Information S2), whereas glutamine enhanced expression of these phosphorylated proteins within 1 h (Table 5, Information S2). Similar results were reported for proline and glutamate (Table 5, Information S2). Phosphorylated PKB, S6K1, and Stat3 were not detectable in the presence of wortmannin or rapamycin.

**Table 4:** Effects of the different dose of amino acids on expression of p-Stat3, p-S6K1, and p-PKB in JAR cells*.

	Concentration of amino acids (mM)							
Items	0	0.25	0.5	1	2	4	SEM	*P* value
Arginine								
p-Stat3/actin	0.05^a^	0.10^b^	0.11^b^	0.14^c^	0.28^d^	0.17^e^	0.002	<0.01
p-S6K1/actin	0.10^a^	0.22^b^	0.16^c^	0.22^b^	0.29^d^	0.26^e^	0.003	<0.01
p-PKB/actin	0.11^a^	0.31^b^	0.30^b^	0.23^c^	0.20^d^	0.22^c^	0.005	<0.01
Glutamine								
p-Stat3/actin	0.10^a^	0.14^b^	0.17^c^	0.28^d^	0.37^e^	0.24^f^	0.005	<0.01
p-S6K1/actin	0.07^a^	0.25^b^	0.18^c^	0.4^d^	0.27^e^	0.19^c^	0.006	<0.01
p-PKB/actin	0.18^a^	0.34^b^	0.51^c^	0.50^d^	0.82^e^	0.71^f^	0.006	<0.01
Glutamate								
p-S6K1/actin	0.15^a^	0.15^a^	0.15^a^	0.14^a^	0.15^a^	0.15^a^	0.004	0.42
p-PKB/actin	0.16^a^	0.16^a^	0.17^a^	0.17^a^	0.17^a^	0.17^a^	0.004	0.21
Proline								
p-S6K1/actin	0.61^a^	0.74^b^	0.63^a^	0.85^c^	0.88^c^	0.84^c^	0.01	<0.01
p-PKB/actin	0.29^a^	0.44^b^	0.44^b^	0.60^c^	0.36^d^	0.30^a^	0.004	<0.01

* Values with different letters are significantly different (*P*<0.05). Values are means ± SEM, n = 6.

**Table 5:** Effects of the different time treatments of amino acids on expression of p-Stat3, p-S6K1, and p-PKB in JAR cells*.

	Time of treatment (h)						
Items	0	0.5	1	2	4	SEM	*P* value
Arginine							
p-Stat3/actin	0.23^a^	0.77^b^	0.76^b^	0.77^b^	0.48^c^	0.004	<0.01
p-S6K1/actin	0.30^a^	0.32^b^	0.36^c^	0.60^d^	0.65^e^	0.004	<0.01
p-PKB/actin	0.10^a^	0.22^b^	0.35^c^	0.25^d^	0.51^e^	0.005	<0.01
Glutamine							
p-Stat3/actin	0.14^a^	0.18^b^	0.20^c^	0.38^d^	0.54^e^	0.004	<0.01
p-S6K1/actin	0.11^a^	0.12^a^	0.22^b^	0.25^c^	0.27^d^	0.005	<0.01
p-PKB/actin	0.18^a^	0.27^b^	0.29^c^	0.23^d^	0.21^e^	0.003	<0.01
Glutamate							
p-S6K1/actin	0.06^a^	0.09^b^	0.08^b^	0.16^c^	0.11^d^	0.004	<0.01
p-PKB/actin	0.10^a^	0.12^b^	0.18^c^	0.43^d^	0.33^e^	0.009	<0.01
Proline							
p-S6K1/actin	0.42^a^	0.76^b^	0.95^c^	0.88^d^	0.86^d^	0.004	<0.01
p-PKB/actin	0.28^a^	0.60^b^	0.55^c^	0.56^c^	0.49^d^	0.005	<0.01

* Values with different letters are significantly different (*P*<0.05). Values are means ± SEM, n = 6.

### LIF expression in Ishikawa cells in response to arginine, glutamine, glutamate, and proline in the presence or absence of LIF antibody

In the absence of LIF antibody, the addition of arginine to the culture medium did not affect LIF expression in Ishikawa cells (Table 6, Information S2). However, glutamine (Information S2), glutamate (Information S2) and proline (Information S2) increased LIF expression in Ishikawa cells (Table 6, *P*<0.05). In response to LIF antibody supplementation, LIF expression in Ishikawa cells was totally inhibited (Table 7, Information S2). Arginine in the culture medium did not affect LIF expression in the presence of LIF antibody (Table 7, Information S2) although glutamine, glutamate and proline increased LIF expression in the presence of LIF antibody (Table 7, Information S2). LIF was not detectable when wortmannin or rapamycin was added to the Ishikawa cell culture medium.

**Table 6:** Effects of the different amino acids on expression of LIF in Ishikawa cells*.

	Time of treatment (h)						
Items	0	0.5	1	2	4	SEM	*P* value
Arginine	0.10^a^	0.10^a^	0.11^a^	0.10^a^	0.11^a^	0.004	0.67
Glutamine	1.02^a^	1.31^b^	1.37^c^	1.26^d^	1.23^e^	0.004	<0.01
Glutamate	0.26^a^	0.55^b^	0.81^c^	0.74^d^	0.74^d^	0.004	<0.01
Proline	0.08^a^	0.27^b^	0.30^c^	0.47^d^	0.49^e^	0.003	<0.01

* Values with different letters are significantly different (*P*<0.05). Values are means ± SEM, n = 6.

**Table 7:** Effects of the different amino acids on the expression of LIF in Ishikawa cells in the presence of LIF antibody*.

	Treatment						
Item	Control	Arginine	Glutamine	Glutamate	Proline	SEM	*P* value
LIF/actin	0^a^	0^a^	0.33^b^	0.10^c^	0.11^c^	0.002	<0.01

* Values with different letters are significantly different (*P*<0.05). Values are means ± SEM, n = 6.

### LIF concentrations in the culture medium of Ishikawa cells in response to arginine, glutamine, glutamate, and proline in the presence or absence of LIF antibody

**Figure 4:**
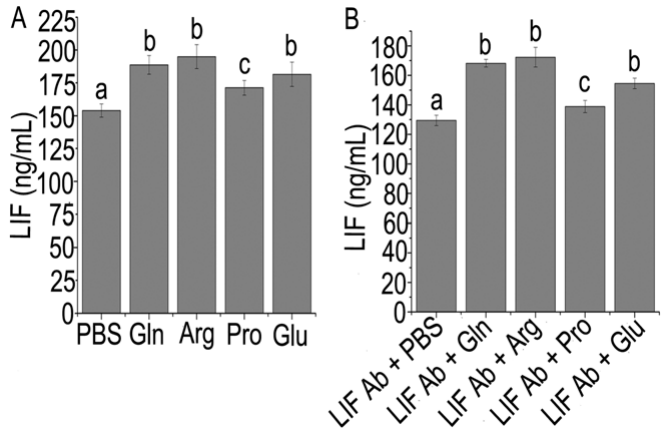
LIF level in medium of Ishikawa cells. LIF concentration in cultural medium of Ishikawa cells supplemented with L-arginine, L-glutamine, L-glutamate, or L-proline in the absence (A) or presence of LIF antibody (B). Data are means ± SEM (n = 6). Means with different letters differ (*P*<0.05). Con, control; Ab, antibody; Arg, arginine; Gln, glutamine; Glu, glutamate; Pro, proline.

In the absence of LIF antibody, arginine, glutamine, glutamate, and proline treatment for 16 h dramatically increased LIF concentration in the culture medium of Ishikawa cells, compared with the control (*P*<0.05, Fig. 4A). Among the four amino acids, the positive effect of arginine was the greatest (Fig. 4A). In the presence of LIF antibody, LIF concentration was still higher in the culture medium of Ishikawa cells supplemented with arginine, glutamine, glutamate, or proline, compared with the control (*P*<0.05, Fig. 4B). In addition, arginine treatment resulted in the greatest positive effect among the four amino acids tested (Fig. 4B).

### Adhesion of JAR cells to fibronectin or laminin in response to arginine, glutamine, glutamate, and proline

**Figure 5:**
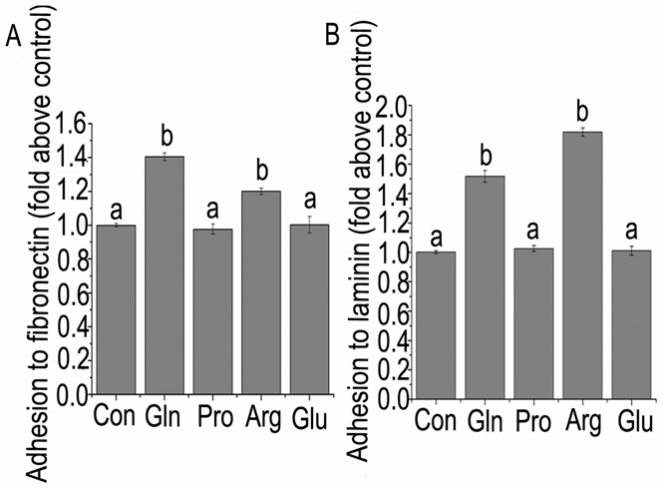
Adhesion rate of JAR cells to fibronectin or laminin. Adhesion of JAR cells to fibronectin (A) or laminin (B) in response to arginine, glutamine, glutamate, and proline supplementation. Data are means ± SEM (n = 6). Means with different letters differ (*P*<0.05). Con, control; Arg, arginine; Gln, glutamine; Glu, glutamate; Pro, proline.

The addition of arginine and glutamine to the culture medium increased the rate of adhesion of JAR cells to fibronectin (Fig. 5A) and laminin (Fig. 5B), compared with controls (*P*<0.05). However, no effect was found for glutamate or proline.

## Discussion

In the present study, we reported that dietary NCG supplementation could increase the litter size and the concentration of arginine family of amino acids in serum and uterine flushings in rats. Besides, we reported dietary NCG supplementation could stimulate PKB/mTOR signaling pathway and LIF expression in rats. Finally, we further confirmed the mechanism in-vitro by using Ishikawa and JAR cells.

Dietary arginine supplementation has been shown to ameliorate intrauterine growth restriction in women [13], [14] and to increase litter size in gilts, sows, and rats [16], [18]. However, arginine is supplied with L-arginine-HCl to animals and humans to prevent acid-base imbalance [25]. With concerns about the effects of chronic provision of chloride on animal and human health [26], as well as the short biological half-life cycle of arginine [27], alternatives should be explored to substitute for arginine. One choice is L-citrulline [25]. On d 135 of gestation, intravenous administration of L-citrulline to pregnant ewes is more effective in increasing concentrations of arginine in both maternal and fetal circulations, compared with arginine [28]. NCG is a metabolically stable activator of arginine synthesis [29] and is not toxic to animals or humans [30], [31]. Oral administration of NCG increases endogenous synthesis of arginine and plasma arginine concentration and muscle protein synthesis in piglets [19]. However, there are no reports of the efficacy of NCG supplementation in pregnant mammals.

In this study, we found that dietary NCG supplementation increased litter size and litter weight. These effects were associated with increases in serum concentrations of arginine, ornithine, and proline and concentrations of arginine, proline, glutamate, and glutamine in uterine flushings. Amino acids are components of uterine fluids which are required for the development of early embryos, and act as precursors of proteins and nucleic acids, energy sources, and signaling molecules [32]. Previous study demonstrated that the amounts of arginine and glutamine in uterine flushings were increased in pregnant ewes during the peri-implantation period [33]. Amino acids are now known to regulate embryo development and blastocyst implantation [34]. Mouse and human embryos express many amino acid transporters to selectively transport arginine [34], suggesting that arginine is important for embryogenesis, embryo/fetal survival, growth, and development, not only for protein synthesis, but also for the production of its metabolites, such as nitric oxide, polyamines, creatine, and prolyl residue-rich extracellular matrix proteins that are important for embryo implantation [35]–[37]. Glutamine is the major energy source for developing conceptuses [38]. Glutamate is important for the synthesis of glutathione, the most abundant antioxidant in cells [39]. During early pregnancy, total glutathione in the uterine fluid of pregnant ewes was also increased [33]. Notably, changes of concentrations of glutamine and glutamate in serum and uterine flushings were not consistent. Although serum concentrations of glutamine and glutamate were not significantly increased, compared with the control group, there was an increase trend. However, the concentrations of these two amino acids in uterine flushings were significantly higher, in comparision with the control group. The reason may be the requirement of these two amino acids for embryo development and implantation resulted in the differences in metabolism in serum and uterine flushings. Thus, increases in concentrations of arginine, proline, glutamate, and glutamine in serum and uterine fluid could supply adequate nutrients for early embryonic development and implantation. These data suggest that dietary NCG supplementation has the potential to improve pregnancy outcomes due to its efficiency in increasing concentrations of the arginine-family of amino acids in maternal circulations and uterine fluids, which could improve early embryonic development and implantation.

Amino acids secreted in the uterine microenvironment are critical to provide nutrients for mammalian embryo development and the regulation of embryo implantation [34], [37]. LIF is an important factor in regulating early embryogenesis and embryo implantation [40], [41]. LIF can enhance the quality of human blastocysts, accelerate embryonic hatching and survival, and increase adhesion of trophoblast cells to fibronectin and laminin [40]–[42]. Female mice lacking LIF produced viable blastocysts, but the blastocysts failed to implant [9]. Exposing embryos to LIF has been shown to improve implantation, pregnancy rate, and viability [43]. To examine whether the increase in the arginine family of amino acids induced by dietary NCG supplementation affects uterine LIF expression, intrauterine administration of LIF antibody was performed on d 4 of pregnancy. Our results indicate that LIF antibody treatment resulted in crowding of embryos, which was reversed by dietary NCG supplementation. In addition, NCG supplementation partially blocked the decline in the number of live fetuses on d 15 of pregnancy induced by LIF antibody treatment. Furthermore, NCG supplementation increased uterine LIF expression regardless of uterine administration of LIF antibody. In order to further determine the contributions of arginine, glutamine, glutamate, and proline (whose concentrations were increased in uterine flushing by dietary NCG supplementation) on uterine LIF expression, Ishikawa cells, a human endometrial epithelial cell line, were used. Although there has been some difficulty in correlating changes observed in Ishikawa cells with the actual changes occurring in vivo in the uterus [44], compared with other cell lines, Ishikawa cell is a good in-vitro model to study embryo implantation [45]. The results showed that glutamine, glutamate, and proline enhanced LIF expression in Ishikawa cells within 4 h and LIF concentration in the culture medium within 16 h in the presence or absence of LIF antibody. Although there was a delayed effect of arginine on LIF expression, this amino acid had the greatest impact on increasing LIF concentrations in the culture medium within 16 h in the presence or absence of LIF antibody. As the purpose of the in vitro experiments is to test the contributions of the four amino acids which were increased in uterine flushings, to exclude the amino acids in the Roswell Park Memorial Institute-1640 (RPMI-1640) culture medium, cells were deprived of amino acids by incubation in Earle's balanced salt solution supplemented with a vitamin mixture. We tried to culture the cells in such medium for 24 hr, and found that there were lots of cells floating in the medium in the presence or absence of the four amino acids' treatment. Also, the attachment of the cells was not as tight as that in the complete RPMI-1640 medium. This may be owing to the nutrition restriction which impaired the cell growth. So finally we decided to culture the cells for no longer than 24 hr. The results indicate that the increase in the arginine family of amino acids induced by dietary NCG supplementation enhanced uterine LIF expression.

To determine the possible signaling pathway mediating the effect of NCG on uterine LIF expression, inhibitors of PI3K and mTOR were administered into the uterine horns. PI3K plays important roles in oocyte maturation, preimplantation embryo development, embryo survival and pregnancy outcome [46]. This cellular signaling pathway is also crucial for placental development as well as differentiation and migration of trophoblast cells [47]–[49]. Inhibition of the PI3K/Akt pathway affected the normal physiology of the blastocyst and delayed blastocyst hatching, ultimately affecting implantation [49]. In addition, inhibition of the PI3K pathway led to the induction of apoptosis in both murine blastocysts and trophoblast stem cells [50]. Inhibition of PI3K results in increased fetal resorption and poor pregnancy outcome [50]. In keeping with these reports, we found that uterine administration of the PI3K inhibitor, wortmannin, almost completely inhibited embryo implantation, and that dietary NCG supplementation could not reverse this effect, indicating that the PI3K signaling pathway is necessary for the action of NCG.

A key downstream target of PI3K is mTOR [51]. The mTOR signaling pathway plays a critical role in embryo implantation. Embryos deficient in mTOR died quickly after implantation owing to the impaired cell proliferation in both the embryonic and extra-embryonic compartments, as well as the impaired ability to form trophoblasts [52], [53]. Amino acid signals could regulate the translation of proteins required for trophoblast differentiation via mTOR-dependent phosphorylation of S6K1 [54]. Intrauterine administration of the mTOR inhibitor, rapamycin, on d 4 of pregnancy decreased implantation sites in mice [55]. Similarly, in our study, rapamycin treatment inhibited embryo implantation in pregnant rats fed the control diet but dietary NCG supplementation attenuated the rapamycin-induced loss of implanted embryos. These data further support the view that the improvement in pregnancy outcome in NCG-supplemented rats requires the stimulation of PI3K/Akt/mTOR signaling pathway. In addition, dietary NCG supplementation increased the expression of uterine LIF and p-Stat3, as well as p-PKB and p-S6K1, even in the presence of LIF antibody. However, the increase in expression of these genes, except p-Stat3, in the NCG group was weak when the uterus received administration of inhibitors of PI3K or mTOR. These findings indicate that the improvement in reproductive performance brought about by dietary NCG supplementation is possibly through the PI3K/Akt/mTOR signaling pathway to stimulate uterine LIF expression. Also there may be other signaling pathways which mediate the role of NCG in regulating LIF, such as nitric oxide, polyamines, or mitogen activated protein kinase (MAPK) pathways, which could be investigated in the future.

Amino acids regulate the development of trophoblast protrusive activity, initiation of motility, and the spreading behaviors which are required for embryo implantation through the mTOR signaling pathway [37]. Additionally, Stat3 is required for murine embryo development [56]. To examine the contributions of arginine, glutamine, glutamate, and proline to the development of trophoblasts *in vitro*, JAR cells, trophoblast-type cells, were used. Results indicated that arginine and glutamine treatment increased the expression of p-PKB, p-S6K1, and p-Stat3 in JAR cells. Proline treatment activated the expression of p-PKB and p-S6K1, but not Stat3. Thus, among the four amino acids tested, both arginine and glutamine stimulated PI3K/Akt/mTOR signaling and Stat3 phosphorylation in trophoblast cells, while proline stimulated the PI3K/Akt/mTOR signaling, but not Stat3 phosphorylation. Furthermore, rapamycin inhibited amino acid stimulation of embryo outgrowth, meaning that mTOR is required for the initiation of protrusive activity in trophoblast cells [54]. In this study, p-PKB, p-S6K1, and p-Stat3 were not detectable in JAR cells treated with arginine, glutamine, glutamate, and proline in the presence of wortmannin or rapamycin. These results indicate that activation of the PI3K/Akt/mTOR signaling is responsible for NCG regulation of Stat3 phosphorylation in JAR cells. Embryos cultured in the amino acid-free mediums could not form trophoblast cell outgrowths on fibronectin, but could do so when transferred into the complete medium [53]. Furthermore, there is evidence that trophoblast invasiveness is connected with Stat3 activity [57]. In this study, we found that arginine and glutamine, but not proline or glutamate, increased the adhesion of JAR trophoblast cells to fibronectin and laminin. The underlying mechanism could be that arginine and glutamine, but not proline and glutamate, increase the expression of p-Stat3. These results demonstrate that among the arginine family of amino acids, both arginine and glutamine may increase the expression of p-Stat3 and adhesiveness of trophoblast cell to the extracellular matrix via activation of the PI3K/Akt/mTOR signaling pathway, which are very important to embryo implantation.

In conclusion, dietary NCG supplementation is effective in increasing the pregnancy outcome in rats because of its efficiency in increasing concentrations of the arginine family of amino acids in maternal circulations and uterine fluids. These amino acids, in turn, may activate the PI3K/Akt/mTOR signaling pathway and Stat3 phosphorylation, stimulate LIF expression, and enhance trophoblast cell adhesion to the extracellular matrix. Such changes may be beneficial to successful embryo implantation and maintenance of pregnancy in mammals. These findings may have important implications for improving the pregnancy outcome of mammalian species.

## Materials and Methods

### Animals and protocols

All rats used in this study were housed and handled according to the established guidelines of the China Department of Agriculture. All procedures were approved by the China Agricultural University Animal Care and Use Committee. The experimental design is summarized in Information S1.

Adult female Sprague-Dawley rats, weighing 220 g to 250 g, were purchased from Beijing Laboratory Animal Center and housed in cages in a temperature (23°C), humidity (relative humidity, 50%), and light (12 h light/12 h dark) controlled room with free access to food and water. Pregnancy was produced by overnight caging of a proestrous female with a fertile male rat. Next morning, the presence of spermatozoa in the vaginal smear was defined as d 1 of pregnancy.

The effects of dietary NCG (Sigma) supplementation during the entire period of pregnancy on pregnancy outcome of rats were determined. A total of 288 pregnant rats was randomly divided into the following 3 dietary groups (n = 96 per treatment group): 1) basal rodent non-purified diet (Science Australia United Efforts Incorporation) [18]; 2) basal rodent non-purified diet plus 0.05% (wt:wt) NCG; and 3) basal rodent non-purified diet plus 0.1% (wt:wt) NCG. At birth, litter size, litter weight, birth weight of pups, the number of dead pups, and the gender of rats were determined.

According to the results, we found 0.1% NCG diet has the most significant effects on the reproductive performance of rats. Therefore, we designed the following animal experiments only including the control and 0.1% NCG groups. To determine the effects of dietary NCG supplementation on the concentrations of amino acids in serum and uterine fluid during early gestation, 30 pregnant rats were assigned randomly to the basal diet or the basal diet plus 0.1% (wt:wt) NCG between d 1 and the evening of d 4, and then food was removed. This dose of NCG was chosen based on the results of the first animal study. Rats received only water until 08:00 on d 5, when all rats were anesthetized by an intraperitoneal injection of sodium pentobarbital (25 mg/kg). Blood samples were collected from the abdominal aorta and serum was stored at −20°C until analysis of amino acids. Uterine flushings were carried out according to the modified methods described by Bonnamy et al.. Briefly, uterine horns of each pregnant rat were exposed by laparotomy, dissected free of connective tissues, clamped at the ends near the cervix. Then 0.9% saline (0.8 mL) was flushed through one horn from the uterotubal junction and the washing recovered at the cervical end of the horn then flushed through the other horn. The collected uterine washing was centrifuged at 3,000×g at 4°C for 15 min and the supernatant was collected and stored at −20°C until amino acid analysis. Thereafter, the uteri were frozen in liquid nitrogen quickly for Western blot analysis.

### Administration of LIF antibody, phosphatidylinositol 3-kinase (PI3K), or mammalian target of rapamycin (mTOR) inhibitors in vivo

To determine whether the effect of dietary NCG supplementation on pregnancy outcome in rats was associated with uterine LIF expression, a series of experiments were conducted by intrauterine injection of LIF antibody. LIF antibody was used as a neutralizing antibody to reduce the total amount of available LIF. Pregnant rats were randomly assigned to either a control or NCG group (n = 12/treatment group) and underwent midventral laparotomy after anesthesia on the morning of day 4 of pregnancy. The fat pad which accompanies ovary and uterus was gently pulled out from the body cavity. LIF antibody (2 µg diluted in 50 µL 0.9% saline; R&D Systems) was slowly injected into the left uterine horn of each rat from the cervix toward the uterotubal junction [58], [59]. Each pregnant rat served as their own control, with the right uterine horn receiving 50 µL 0.9% saline. On d 7 of pregnancy, six rats in each group were killed after anesthesia. Uteri were frozen in liquid nitrogen quickly after the number of implantation sites was counted in each uterine horn. The remaining 6 pregnant rats in each group were killed on d 15 of pregnancy after anesthesia. The weights of conceptuses, fetuses, placentas, and the number of live fetuses were recorded.

To establish whether the PI3K/protein kinase B (PKB)/mTOR signaling transduction pathway is involved in the enhancement of pregnancy outcome by NCG, we investigated the effects of blocking these signaling molecules by using intrauterine injection of their inhibitors. Pregnant rats were randomly assigned to either the control or NCG group. The left uterine horn of all rats underwent intrauterine injection of 20 ug wortmannin (inhibitor of PI3K, diluted in 50 µL 0.9% saline; n = 12; Sigma) or 20 ug rapamycin (inhibitor of mTOR, diluted in 50 µL 0.9% saline; n = 14; Sigma) on d 4 of pregnancy [60], [61]. The right uterine horn was injected with saline as the control. All rats were killed after anesthesia on d 7 of pregnancy. Uteri were sampled as above after the number of implanted embryos was recorded.

### Cell culture and treatment

To further examine the contributions of arginine, glutamine, glutamate, and proline, which were increased in uterine flushings of rats fed the NCG-supplemented diet, Ishikawa cells and JAR cells were used as *in vitro* models. We initially tried to isolate endometrial cells from pregnant rates (day 3) according to the procedures reported by Arnold and colleagues [62]. Unfortunately, the density and purity of the isolated endometrial cells, harvested from the rat uteri, were not acceptable for subsequent in vitro experiments. Ishikawa cells (human endometrial epithelial cell line) and JAR cells (human trophoblastic cell line) were obtained from European Collection of Cell Culture (ECACC) and American Type Culture Collection (ATCC) and cultured in RPMI-1640 (Hyclone) medium supplemented with 10% fetal bovine serum (FBS). After reaching confluence, cells were deprived of amino acids by incubation in Earle's balanced salt solution (EBSS) (Sigma) supplemented with a vitamin mixture (catalog No. R7256, Sigma), according to the established protocols [63]. After 3 h, cells were used for the following experiments.

JAR cells were cultured in EBSS medium supplemented with 0, 0.25, 0.5, 1.0, 2.0, and 4.0 mM arginine, glutamine, glutamate, or proline (Sigma). After 2 h, cells were collected to detect the expression of p-PKB, p-S6K1, and p-Stat3. The time course (0, 0.5, 1.0 2.0, and 4.0 h) of the change in the expression of p-PKB, p-S6K1, and p-Stat3 in the presence of 2 mM of either arginine, glutamine, glutamate, or proline in the culture of JAR cells was determined. In addition, JAR cells were incubated in EBSS medium containing wortmannin (10 µM) or rapamycin (20 nM). After 30 min incubation, phosphate buffered saline (PBS) or 2 mM of arginine, glutamine, glutamate or proline was added to the medium for each inhibitor treatment. After 2 h, cells were harvested to detect p-PKB, p-S6K1, and p-Stat3.

Ishikawa cells were cultured in EBSS medium supplemented with PBS or 2 mM arginine, glutamine, glutamate, or proline. After 2 h, the expression of LIF in cells was determined. Additionally, Ishikawa cells were incubated in EBSS medium containing 2 µg LIF antibody plus PBS or 2 mM arginine, glutamine, glutamate, or proline. After 2 h, cells were harvested to detect LIF expression. Ishikawa cells also were incubated in EBSS medium containing wortmannin (10 µM) or rapamycin (20 nM). After a 30 min incubation period, PBS or 2 mM of arginine, glutamine, glutamate or proline for each inhibitor treatment were added. After 2 h, cells were harvested to detect LIF expression. Ishikawa cells also were cultured in EBSS medium supplemented with PBS or 2 mM arginine, glutamine, glutamate or proline, in the presence or absence of 2 µg LIF antibody. After 16 h, medium was collected for LIF assay following the manufacturer's instruction (R&D Systems).

### Cell adhesion assay

To investigate the contributions of arginine, glutamine, glutamate, and proline on the adhesion of trophoblast cells to different extra-cellular matrix elements, the adhesion of JAR cells to fibronectin or laminin was performed *in vitro* using fibronectin or laminin-coated CytoMatrix cell adhesion strips (Millipore). Briefly, cells were starved as indicated above, and then treated with PBS or 2 mM arginine, glutamine, glutamate, or proline for 2 h. After that, 100 µL of the diluted cell suspension (1×10^6^ cells/mL) was seeded to wells coated with fibronectin or laminin. The plate was incubated at 37°C for 1 h in a CO_2_ incubator. Wells were gently washed 2–3 times with PBS containing Ca^2+^/Mg^2+^ (200 µL/well) followed by addition of 0.2% crystal violet in 10% ethanol and incubation for 5 min at room temperature. The stain was removed, and the wells gently washed. Cells were solubilized by addition of 100 µL of solubilization buffer (a 50/50 mixture of 0.1 M NaH_2_PO_4_, pH 4.5 and 50% ethanol). The absorbance was determined at 570 nm on a microplate reader.

### Western blot analysis

Cells or frozen uteri were homogenized in the RIPA lysis buffer containing protease inhibitor cocktails (Amresco). After 30 min incubation, homogenates were centrifuged at 14,000× g for 15 min at 4°C, the supernatant fluid collected, and stored at −80°C. Protein concentrations were determined by using the BCA protein assay kit (Pierce). Equal amounts of proteins (35 µg total protein for LIF, p-PKB, p-S6K1, p-Stat3, and β-actin) were electrophoresed (Bio-Rad) on SDS-polyacrylamide gels. Proteins were electro-transferred to a PVDF membrane (Millipore) and blocked with 5% nonfat dry milk at 4°C overnight. Prestained protein markers (Fermentas) were run in each gel. Samples were incubated with rabbit anti-rat polyclonal antibodies (1∶1000 dilution for 2 h at room temperature or overnight at 4°C) against LIF, p-PKB (Ser473), p-S6K1 (Thr389), p-Stat3 (Tyr705), or β-actin (anti-LIF, R&D Systems; anti-p-S6K1, and anti-β-actin, Santa Cruz; anti-p-PKB, anti-p-Stat3, Cell Signaling Technology). After being washed with Tris-Tween-20 buffer (pH 7.4), membranes were incubated with the horseradish peroxidase-conjugated goat anti-rabbit IgG (ZSGB-BIO) for 45 min at room temperature. The membrane was exposed to the X-ray film for 1 to 6 min. Band densities were detected with the western blotting luminance reagent (Santa Cruz) and quantified using AlphaImager 2200 (Alpha Innotech) software.

### Statistical analysis

Data of the reproductive performance and cell culture experiments were subjected to one-way analysis of variance (ANOVA), followed by Duncan's Multiple Range test using the General Linear Model (GLM) procedures of SAS Statistical Software (Version 9, SAS Institute). Data on amino acids in serum and uterine flushing were analyzed using the t-test of SAS (version 8.0, SAS Institute). Results are expressed as means ± SEM. *P*<0.05 was considered significant.

## Supporting Information

Information S1 **Supporting tables.** Table S1. Effects of dietary NCG supplementation on the concentrations of amino acids in serum. Table S2. Effects of dietary NCG supplementation on the concentrations of amino acids in uterine flushings of the pregnant rats. Table S3. Summary of the experimental designs in this study.
(DOC)

Information S2 **Supporting figures.** Figure S1. Expression of uterine p-Stat3, LIF, p-S6K1, and p-PKB in rats. Rats were fed a NCG-supplemented or control diet on d 5 (A), and d 7 of pregnancy in the presence of LIF antibody (B), and on d 7 in the presence of wortmannin (C) and rapamycin (D). Ab = antibody, Wort = wortmannin, Rapa = rapamycin. Figure S2. Dose response and time course for expression of p-Stat3, p-S6K1, and p-PKB in JAR cells. Cells were supplemented with L-arginine (A, B), L-glutamine (C, D), L-glutamate (E, F), or L-proline (G, H). Arg, arginine; Gln, glutamine; Glu, glutamate; Pro, proline. Figure S3. Expression of LIF in Ishikawa cells. Cells were supplemented with L-arginine (A), L-glutamine (B), L-glutamate (C), or L-proline (D) for 0, 0.5, 1.0, 2.0, and 4.0 h or supplemented with 2 µg LIF antibody plus L-arginine, L-glutamine, L-glutamate or L-proline (E). Ab, antibody; Arg, arginine; Gln, glutamine; Glu, glutamate; Pro, proline.
(DOC)
