# Supplementary material for: N-Carbamylglutamate Enhances Pregnancy Outcome in Rats through Activation of the PI3K/PKB/mTOR Signaling Pathway
Source: PLoS One. 2012 Jul 27;7(7):e41192. doi: 10.1371/journal.pone.0041192 (PMC3407155; doi:10.1371/journal.pone.0041192)
Supplement: Information S1 — Supporting tables. Table S1. Effects of dietary NCG supplementation on the concentrations of amino acids in serum. Table S2. Effects of dietary NCG supplementation on the concentrations of amino acids in uterine flushings of the pregnant rats. Table S3. Summary of the experimental designs in this study. (DOC) [file pone.0041192.s001.doc]

**Table S1.** Effects of dietary NCG supplementation on the concentrations of amino acids in serum of the pregnant rats^*^

| Items | Concentrations of amino acids in Serum（μM） | | *P* value |
| --- | --- | --- | --- |
|  | Control | 0.1% NCG |  |
| N | 15 | 15 |  |
| Phosphoserine | 89.4 ± 2.5 | 87.4 ± 3.1 | 0.63 |
| Taurine | 319.7 ± 19.25 | 323.8 ± 17.85 | 0.88 |
| Aspartic acid | 7.0 ± 0.61 | 8.4 ± 0.94 | 0.22 |
| Threonine | 392.2 ± 22.92 | 431.6 ± 29.36 | 0.29 |
| Serine | 292.3 ± 10.13 | 319.2 ± 16.47 | 0.18 |
| Asaparagine | 166.3 ± 8.63 | 181.1 ± 7.79 | 0.21 |
| Glycine | 293.4 ± 10.76 | 298.9 ± 14.31 | 0.76 |
| Alanine | 520.7 ± 14.3 | 533.8 ± 31.23 | 0.71 |
| Citrulline | 60.7 ± 2.54 | 63.3 ± 3.26 | 0.54 |
| Valine | 196.3 ± 6.4 | 184.2 ± 6.96 | 0.21 |
| Methionine | 63.6 ± 1.67 | 67.7 ± 2.17 | 0.14 |
| Isoleucine | 97.7 ± 4.14 | 95.2 ± 4.89 | 0.70 |
| Leucine | 168.2 ± 6.36 | 158.0 ± 6.16 | 0.26 |
| Tyrosine | 76.0 ± 3.45 | 73.1 ± 3.96 | 0.58 |
| Phenylalanine | 66.4 ± 3.12 | 61.4 ± 2.30 | 0.21 |
| Histidine | 46.7 ± 1.52 | 46.9 ± 1.1 | 0.93 |
| Tryptophane | 97.9 ± 6.84 | 90.5 ± 4.24 | 0.36 |
| Lysine | 568.8 ± 21.61 | 576.1 ± 22.32 | 0.81 |

^*^Data are means ± SEM.

**Table S2. Effects of dietary NCG supplementation on the concentrations of amino acids in uterine flushings of the pregnant rats^*^**

| Items | Concentrations of amino acids in uterine flushings（μM） | | *P* value |
| --- | --- | --- | --- |
|  | Control | 0.1% NCG |  |
| N | 15 | 15 |  |
| Phosphoserine | 12.2 ± 0.34 | 12.9 ± 0.75 | 0.43 |
| Taurine | 94.4 ± 6.23 | 96.1 ± 5.16 | 0.84 |
| Aspartic acid | 0.35 ± 0.02 | 0.34 ± 0.03 | 0.79 |
| Threonine | 10.1 ± 0.78 | 11.7 ± 0.88 | 0.19 |
| Serine | 8.4 ± 0.62 | 9.6 ± 0.8 | 0.23 |
| Asaparagine | 8.5 ± 0.43 | 9.2 ± 0.54 | 0.34 |
| Glycine | 22.7 ±. 0.98 | 25.0 ± 2.16 | 0.33 |
| Alanine | 17.1 ± 0.86 | 19.5 ± 1.51 | 0.17 |
| Citrulline | 1.33 ± 0.08 | 1.4 ± 30.1 | 0.59 |
| Valine | 3.3 ± 0.28 | 3.6 ± 0.31 | 0.47 |
| Methionine | 0.74 ± 0.08 | 0.89 ± 0.10 | 0.26 |
| Isoleucine | 1.66 ± 0.15 | 1.84 ± 0.16 | 0.43 |
| Leucine | 3.5 ± 0.27 | 4.1 ± 0.31 | 0.15 |
| Tyrosine | 1.95 ± 0.21 | 1.73 ± 0.18 | 0.42 |
| Phenylalanine | 1.42 ± 0.10 | 1.43 ± 0.13 | 0.96 |
| Histidine | 1.2 ± 0.13 | 1.2 ± 0.10 | 0.98 |
| Tryptophane | 0.78 ± 0.07 | 0.86 ± 0.15 | 0.59 |
| Lysine | 5.7 ± 0.51 | 6.9 ± 0.71 | 0.19 |

^*^Data are means ± SEM.

**Table S3 Summary of the experimental designs in this study**

| Items | Dietary treatments | Length of treatment | Sample size |
| --- | --- | --- | --- |
| For reproductive performance | 0, 0.05%, 0.1% NCG | The whole pregnancy period | n = 96 for each treatment |
| For concentrations of amino acids in serum or uterine flushing | 0, 0.1% NCG | Between d 1 and d 4 | n = 15 for each treatment |
| For intrauterine injection of LIF antibody | 0%, 0.1% NCG | Between d 1 and d 15. | n = 12 for each treatment |
| For intrauterine injection of wortmannin | 0%, 0.1% NCG | Between d 1 and d 7. | n = 6 for each treatment |
| For intrauterine injection of rapamycin | 0%, 0.1% NCG | Between d 1 and d 7. | n = 7 for each treatment |
| JAR cells | Treatments | | |
| Dose response | Culture cells with 0, 0.25, 0.5, 1.0, 2.0, and 4.0 mM arginine, glutamine, glutamate, or proline for 2 h. | | |
| Time course | Culture cells with 2 mM of either arginine, glutamine, glutamate, or proline for 0, 0.5, 1.0 2.0, and 4.0 h. | | |
| Inhibitors’ treatments | Culture cells with wortmannin (10 μM) or rapamycin (20 nM) for 0.5 h, then culture with PBS or 2 mM of arginine, glutamine, glutamate or proline for 2 h. | | |
| Cell adhesion | Culture cells with PBS or 2 mM of arginine, glutamine, glutamate, or proline for 2 h. Then cells were used to measure the adhesion of cells to fibronectin or laminin. | | |
| Ishikawa cells | Treatments | | |
| LIF expression in Ishikawa cells | Culture cells with PBS or 2 mM arginine, glutamine, glutamate, or proline for 2 h. | | |
| LIF expression in Ishikawa cells in the presence of LIF antibody | Culture cells with 2 µg LIF antibody plus PBS or 2 mM arginine, glutamine, glutamate, or proline for 2 h. | | |
| LIF level in culture medium | Culture cells with PBS or 2 mM arginine, glutamine, glutamate, or proline for 16 h. | | |
| Inhibitors’ treatments | Culture cells with wortmannin (10 μM) or rapamycin (20 nM) for 0.5 h, then culture with PBS or 2 mM of arginine, glutamine, glutamate or proline for 2 h. | | |
