# Supplementary figures and images for: N-Carbamylglutamate Enhances Pregnancy Outcome in Rats through Activation of the PI3K/PKB/mTOR Signaling Pathway
Source: PLoS One. 2012 Jul 27;7(7):e41192. doi: 10.1371/journal.pone.0041192 (PMC3407155; doi:10.1371/journal.pone.0041192)

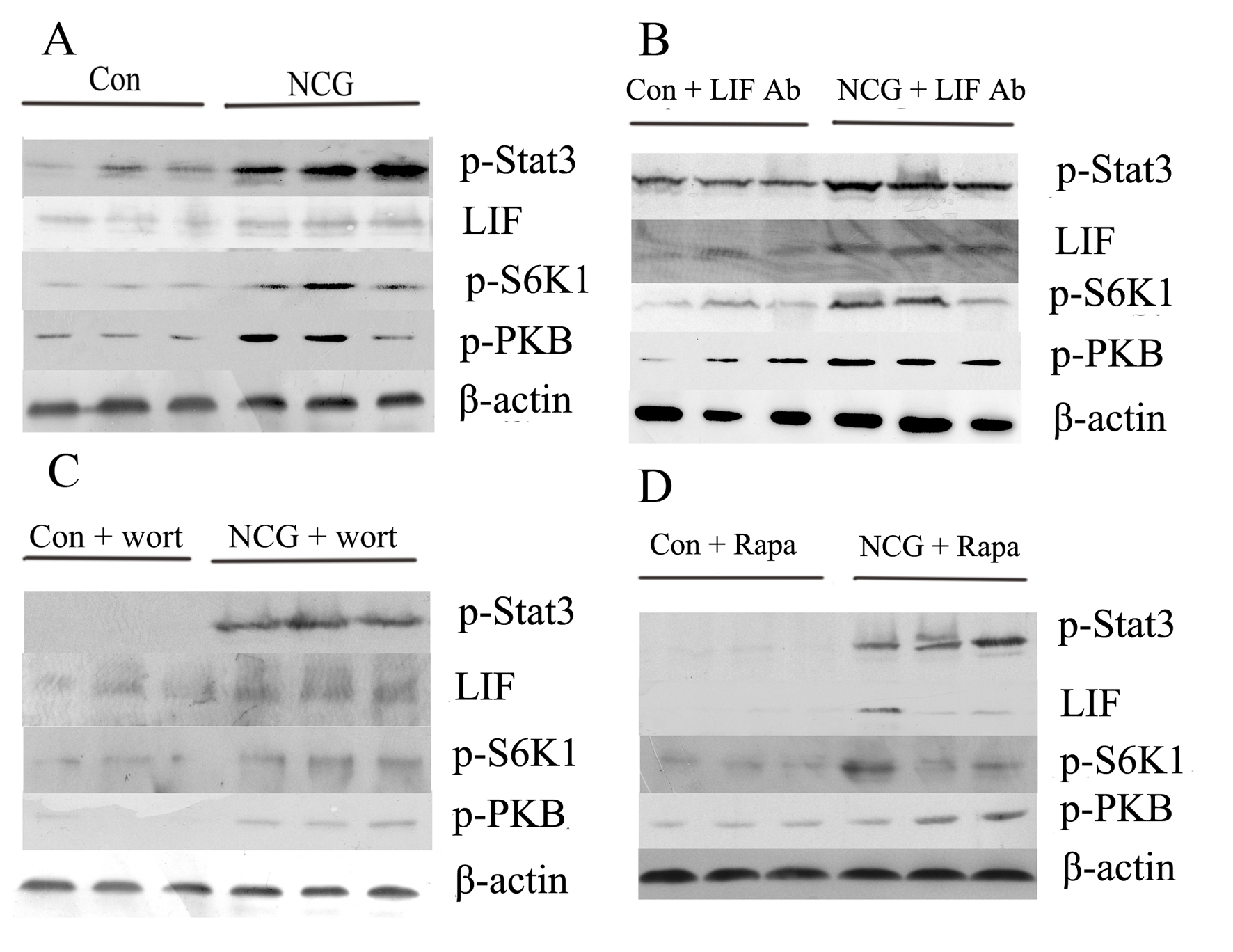


Figure S1

**
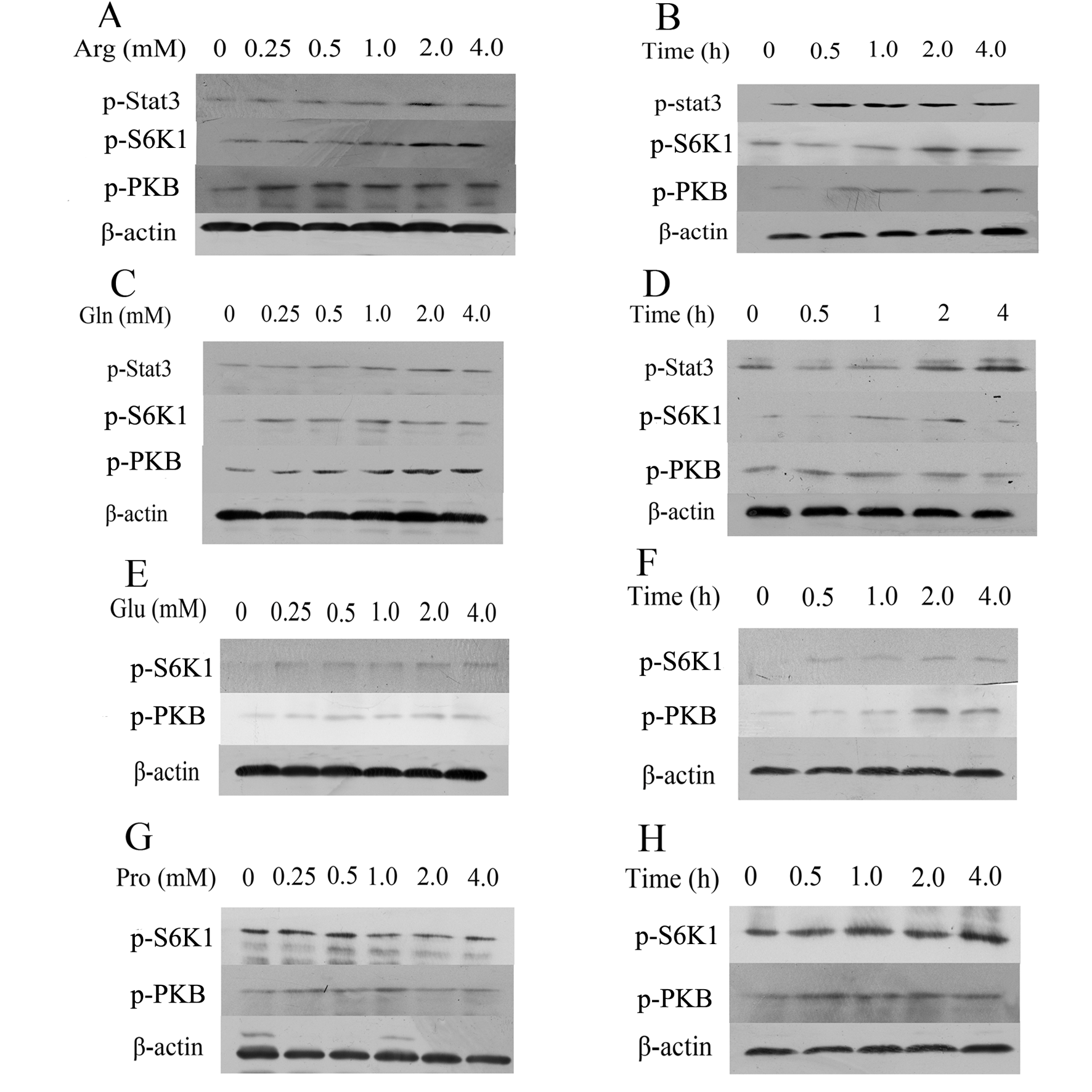
**

Figure S2


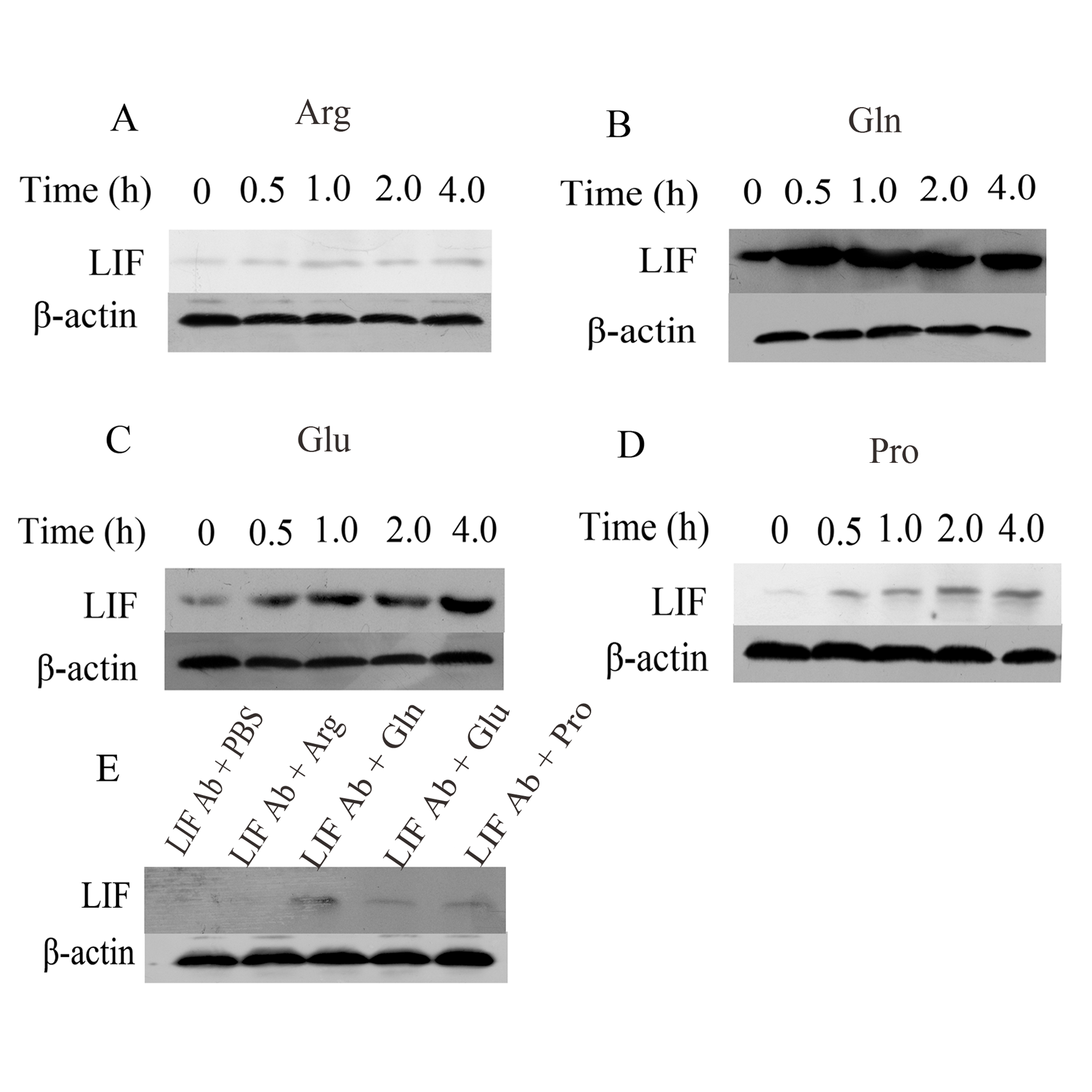


Figure S3

Supplement: Information S2 — Supporting figures. Figure S1. Expression of uterine p-Stat3, LIF, p-S6K1, and p-PKB in rats. Rats were fed a NCG-supplemented or control diet on d 5 (A), and d 7 of pregnancy in the presence of LIF antibody (B), and on d 7 in the presence of wortmannin (C) and rapamycin (D). Ab = antibody, Wort = wortmannin, Rapa = rapamycin. Figure S2. Dose response and time course for expression of p-Stat3, p-S6K1, and p-PKB in JAR cells. Cells were supplemented with L-arginine (A, B), L-glutamine (C, D), L-glutamate (E, F), or L-proline (G, H). Arg, arginine; Gln, glutamine; Glu, glutamate; Pro, proline. Figure S3. Expression of LIF in Ishikawa cells. Cells were supplemented with L-arginine (A), L-glutamine (B), L-glutamate (C), or L-proline (D) for 0, 0.5, 1.0, 2.0, and 4.0 h or supplemented with 2 µg LIF antibody plus L-arginine, L-glutamine, L-glutamate or L-proline (E). Ab, antibody; Arg, arginine; Gln, glutamine; Glu, glutamate; Pro, proline. (DOC) [file pone.0041192.s002.doc]
